# Insights into the cellulose degradation mechanism of the thermophilic fungus *Chaetomium thermophilum* based on integrated functional omics

**DOI:** 10.1186/s13068-020-01783-z

**Published:** 2020-08-12

**Authors:** Xin Li, Chao Han, Weiguang Li, Guanjun Chen, Lushan Wang

**Affiliations:** State Key Laboratory of Microbial Technology, Microbial Technology Institute, Shandong University, No. 72 Jimo Binhai Road, Qingdao, 266237 Shandong People’s Republic of China

**Keywords:** Lytic polysaccharide monooxygenase, *Chaetomium thermophilum*, Thermophilic cellulase, Lignocellulosic enzyme system, Crystalline cellulose

## Abstract

**Background:**

Lignocellulose is the most abundant and renewable biomass resource on the planet. Lignocellulose can be converted into biofuels and high-value compounds; however, its recalcitrance makes its breakdown a challenge. Lytic polysaccharide monooxygenases (LPMOs) offer tremendous promise for the degradation of recalcitrant polysaccharides. *Chaetomium thermophilum*, having many LPMO-coding genes, is a dominant thermophilic fungus in cellulose-rich and self-heating habitats. This study explores the genome, secretomes and transcript levels of specific genes of *C. thermophilum*.

**Results:**

The genome of *C. thermophilum* encoded a comprehensive set of cellulose- and xylan-degrading enzymes, especially 18 AA9 LPMOs that belonged to different subfamilies. Extracellular secretomes showed that arabinose and microcrystalline cellulose (MCC) could specifically induce the secretion of carbohydrate-active enzymes (CAZymes), especially AA9 LPMOs, by *C. thermophilum* under different carbon sources. Temporal analyses of secretomes and transcripts revealed that arabinose induced the secretion of xylanases by *C. thermophilum*, which was obviously different from other common filamentous fungi. MCC could efficiently induce the specific secretion of LPMO2s, possibly because the insert in loop3 on the substrate-binding surface of LPMO2s strengthened its binding capacity to cellulose. LPMO2s, cellobio hydrolases (CBHs) and cellobiose dehydrogenases (CDHs) were cosecreted, forming an efficient cellulose degradation system of oxidases and hydrolases under thermophilic conditions.

**Conclusions:**

The specific expression of LPMO2s and cosecretion of hydrolases and oxidases by the thermophilic fungus *C. thermophilum* play an important role in cellulose degradation. This insight increases our understanding of the cellulose degradation under thermophilic conditions and may inspire the design of the optimal enzyme cocktails for more efficient exploration of biomass resources in industrial applications.

## Background

Lignocellulose is the most abundant, renewable and sustainable biomass resource on the planet and is composed primarily of three polymer components: cellulose, hemicellulose and lignin [1]. Cellulose is the most abundant polysaccharide and is composed of repeating β-1,4-d-glucose units [2]. Multiple cellulose chains form an elementary cellulose fibril, and many fibrils pack into an insoluble crystalline structure that is stable and insoluble. Moreover, xylan, which covers cellulose microfibrils, is complex and heterogeneous, further restricting cellulases accessibility [3, 4]. Enzymatic deconstruction of these components is a major bottleneck for the efficient transformation of cellulose.

In the long process of evolution, microorganisms have evolved the ability to generate several lignocellulosic-degrading enzymes, including glycoside hydrolases (GHs) and oxidases [5, 6]. Cellulolytic enzymes include exoglucanases (CBHs, EC 3.2.1.91/176), endoglucanases (EGs, EC 3.2.1.4) and β-glucosidases (BGs, EC 3.2.1.21) [7]. The classical paradigm of cellulose degradation by hydrolytic enzymes, which has been valid for decades, was revisited by the discovery of lytic polysaccharide monooxygenases (LPMOs) [8, 9]. LPMOs can catalyze the regioselective hydroxylation of polysaccharides, leading to glycosidic bond cleavage in the presence of copper ions, reducing agents and O_2_ [10]. Studies have reported that LPMOs contribute to the enhancement of biomass degradation and reduce the amount of glycoside hydrolase loading in practice [11–14]. Therefore, a new research focus was formed in the field of lignocellulose degradation and transformation.

The carbohydrate-active enzymes database (CAZy, http://www.cazy.org/) expanded rapidly with the development of omics technology. Data related to LPMO also quickly increased. Therefore, the CAZy database launched a new CAZy class, named auxiliary activities (AAs), which combines the categories of LPMOs and lignin-degrading enzymes [15]. To date, more than 13,000 sequences have been collected. LPMOs are mainly distributed in families AA9, AA10, AA11, AA13, AA14, AA15 and AA16 and can act on a variety of polysaccharide substrates such as cellulose and chitin [16–20]. Family AA9 is the main LPMO in fungi, with more than 500 sequences. Most thermophilic LPMOs, which have high biotechnological potential, are characterized in the family AA9 [21]. AA9 LPMOs were further divided into three subfamilies based on sequence similarity, including LPMO1, LPMO2 and LPMO3 [17]. LPMO1, LPMO2, and LPMO3 hydroxylate the glycosidic bond at position C1, C4, C1/C4 when acting on polysaccharide substrates, respectively [17]. In addition, the specificity of AA9 LPMOs for their substrates is wide, including cellulose, cello-oligosaccharide and β-1,4-linked hemicelluloses [17, 22–26]. Furthermore, hydroxylation is more likely to occur at glycosidic position C6 for substrates such as soluble oligosaccharides [27]. The substrate-binding surface formed by longer loops (L2, L3, LC, LS, etc.) is different among members in AA9 subfamily. These loops and solvent-exposed aromatic residues that located on them are believed to influence regioselectivity, substrate recognition and specificity. For example, the NCU07760 (LPMO3) mutant lacking an insert of L2 could not hydroxylate the glycosidic bond at position C4, indicating that this insert is important for C4 selectivity [17]; altering the aromatic residues on the substrate-binding surface can alter C1/C4-oxidation ratios, such as Y24 of L2 and Y 211 of LC have been shown to have an effect on the regioselectivity of *Hj*LPMO9A [28]. Due to the complexity and rapid electron transfer of the reaction system, the reaction kinetics of AA9 LPMOs have not been well established; thus, further studies are needed to clarify the exact physiological function and the optimal natural substrate of AA9 subfamilies, especially under thermophilic conditions.

The rapid development of genome sequencing and proteomics technology has provided a new perspective on the study of AA9 LPMOs. Genes encoding AA9 LPMOs not only are widely distributed in fungi, but also have significantly expanded in some fungal species [29]. The genome of *Pycnoporus coccineus* encodes 15 candidate AA9 LPMOs, but species of AA9 detected in the proteomes of *P. coccineus* were different when cultured with different substrates [30, 31], which may be related to the substrate composition. AA9 LPMOs combined with cellulases can effectively improve the conversion rate of substrates and reduce enzyme loading. Enzyme cost is a necessary consideration in industrial applications [12]; therefore, many efforts have been made to design better commercial enzyme cocktails. However, the enzymes necessary for efficient synergy and their efficiency are still unclear, especially under thermophilic conditions. Mesophilic enzymes have a low performance under thermophilic conditions, which are common in industrial applications [32]; therefore, enzyme stability is also an important factor. Thermophilic enzymes have attracted extensive attention due to their good stability and low cost in production and application processes and mainly come from thermophilic fungi [21]. The thermophilic enzyme has become one of the hot spots in the development of industrial enzyme cocktails.

*Chaetomium thermophilum* is a thermophilic fungus with an optimum growth temperature of 50 °C. It has been suggested as a model organism for structural and biochemical analyses of functional proteins [33, 34]. It is also widely distributed in self-heating and cellulose-rich habitats and can efficiently degrade crystalline cellulose [35]. The whole genome of *C. thermophilum* DSM 1495 was sequenced in 2011 [33]. Here, we provide insights into the cellulose degradation mechanism of the thermophilic fungus *C. thermophilum* CGMCC3.17990. By integrating biochemical assays, functional secretomics and transcription analysis of specific genes, we analyzed the substrate-dependent and temporal changes of the extracellular CAZymes secreted by this fungus, especially for AA9 LPMOs under thermophilic conditions.

## Results

### *Chaetomium thermophilum* encodes an efficient polysaccharide-degrading enzyme system

*Chaetomium thermophilum* is widely distributed in self-heating and cellulose-rich habitats; therefore, enzymes related to biomass degradation were selected from the genome of *C. thermophilum*. *C. thermophilum* encoded 298 CAZymes, including 64 AAs, 136 GHs, 25 carbohydrate esterases (CEs), 4 polysaccharide lyases (PLs) and 69 glycoside transferases (GTs) (Additional file 1: Table S1). Furthermore, 51, 28, 24, 18 and 11 genes were related to the degradation of cellulose, xylan, β-1,3;1,4-glucan, chitin and starch, respectively (Table 1). However, few genes encoded enzymes that degraded xyloglucan, galactomannan and pectin (Table 1). The cell walls of dicots (grasses) mainly include cellulose, xyloglucan and pectin, but the cell walls of monocots mainly consist of cellulose, glucuronoarabinoxylan and mixed linkage β-glucan [36]. Therefore, it was suggested that *C. thermophilum* could degrade monocots better than dicots. In addition, β-1,3;1,4-glucan and chitin are abundant in the cell wall of fungi [37]. As a result, *C. thermophilum* was thought to be capable of deconstructing the cell wall of fungi.

**Table 1:** Biomass-degrading enzymes in the genome of *C. thermophilum*

Type	Substrate	Enzyme activity	CAZy family	Number
Oxidases	Lignocellulose	Lytic polysaccharide monooxygenase	AA9	18
		Cellobiose dehydrogenase	AA3+AA8	2
		GMC oxidoreductase	AA3	8
		Oligosaccharide oxidase	AA7	13
		Copper radical oxidases	AA5	8
		Iron reductase	AA8	5
		Multicopper oxidase	AA1	5
		Class II lignin-modifying peroxidases	AA2	2
		Vanillyl-alcohol oxidase	AA4	2
		1,4-Benzoquinone reductase	AA6	1
Non-oxidases	Cellulose	Cellobiohydrolase	GH7	3
			GH6	2
		β-1,4-Glucosidase	GH3	7
			GH1	1
		β-1,4-Endoglucanase	GH5	5
			GH45	2
			GH6	1
			GH7	1
			GH12	1
	Xylan	β-1,4-Endoxylanase	GH10	4
			GH11	4
		β-1,4-Xylosidase	GH43	3
		Acetylxylan esterase	CE3	4
			CE1	2
			CE5	2
		α-Glucuronidase	GH67	1
			GH115	1
		Feruloyl esterase	CE1	2
		α-Arabinofuranosidase	GH43	3
			GH62	2
	(Galacto)mannan	β-1,4-Endomannase	GH5	1
			GH26	1
		β-1,4-Mannosidase	GH2	1
	Xyloglucan	β-1,4-Endoglucanase	GH74	1
	Pectin	Pectate lyase	PL3	2
			PL1	1
			PL2	1
		Exopolygalacturonase	GH28	1
		β-1,4-Galactosidase	GH2	1
			GH35	1
		Pectin methylesterase	CE8	1
		Carbohydrate esterase	CE15	2
			CE16	2
		Rhamnogalacturonan acetylesterase	CE12	1
	β-1,3;1,4-glucan	1,3-β-Endoglucanase	GH16	11
			GH17	3
			GH81	1
			GH55	1
			GH128	2
			GH131	2
		1,3-β-Exodoglucanase	GH55	3
			GH132	1
	Chitin	Exo-β-1,4-d-glucosaminidase	GH2	1
		Chitinase	GH18	9
			GH75	1
		β-Hexosaminidase	GH20	1
		Chitin-binding protein	CBM18	2
		Deacetylase	CE4	4
	Starch	α-Amylase	GH13	4
		α-Glucosidase	GH31	4
			GH15	3

**Fig. 1:**
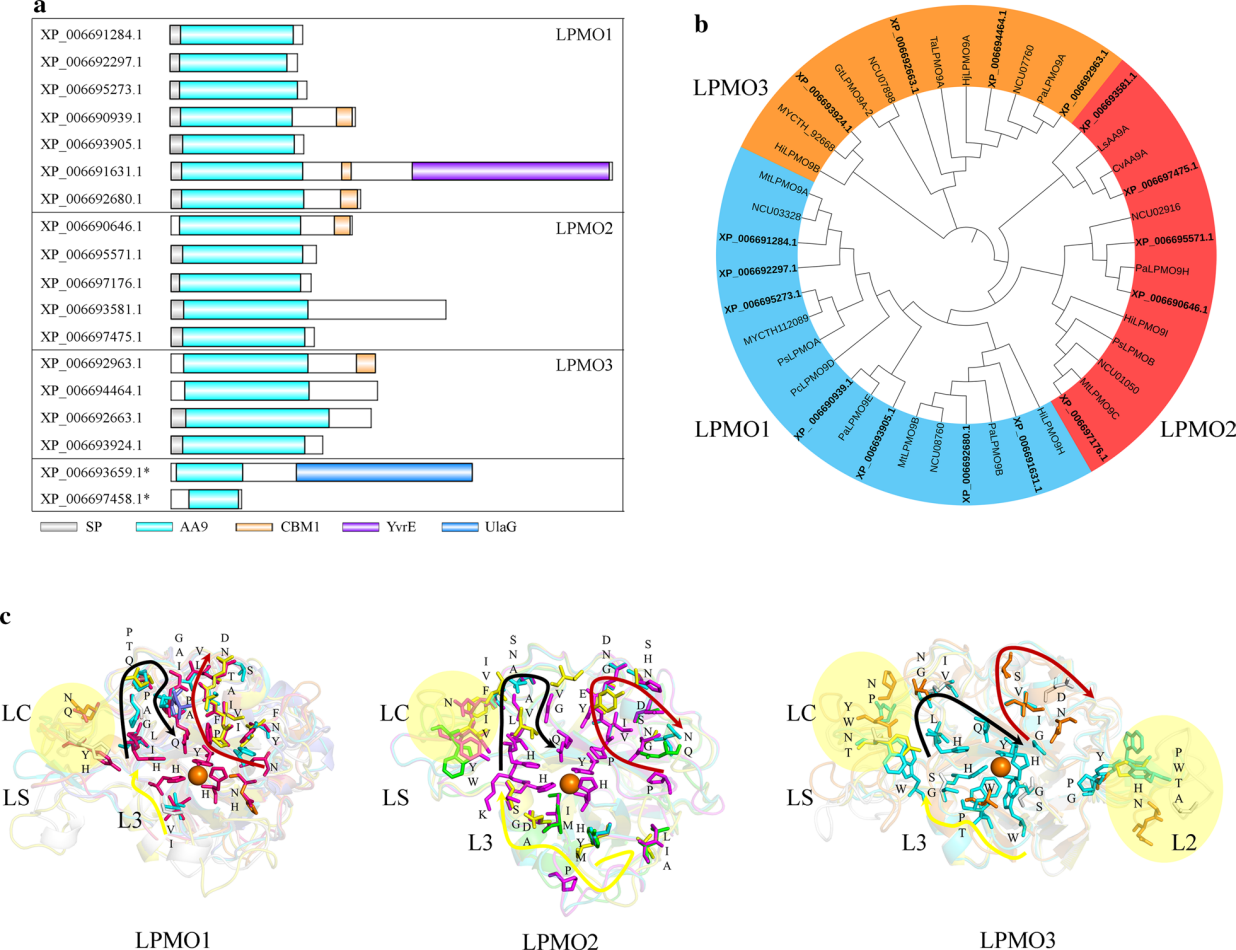
Bioinformatics analysis of AA9 LPMOs of *C. thermophilum* DSM 1495. **a** Domain distributions of AA9 LPMOs. The figure shows the schematic of the arrangement of domains in each AA9 LPMO. **b** Phylogenetic tree of AA9 LPMOs. LPMO1, LPMO2 and LPMO3 are shown in blue, red and orange, respectively. Labels of AA9 LPMOs from *C. thermophilum* are bold, and the other labels are not bold. The sequences of the AA9 catalytic domains in XP_006693659.1 and XP_006697458.1 are too short to align, so they were deleted in the phylogenetic tree. **c** The substrate-binding surface and its conserved residues in the modeled structures of the AA9 subfamily. This figure shows a top view of structurally aligned and superimposed AA9 LPMOs grouped according to the clusters defined in **b**. Residues protruding from the surface are shown as sticks and labeled. Three regions (depicted by black, red and yellow arrows) define the immediate environment of the catalytic center, and the region of yellow arrow is the loop3 (L3, the loop between β-strands 3 and 4). Additional conspicuous surface residues more remote from the catalytic centers appear in the yellow-shaded areas that are formed by additional residues in the loop2 (L2, the loop between β-strands 1 and 2) region (only in LPMO3) and/or the LS/LC loops (LS, loop short; LC, long C-terminal loop)

The cellulose-degrading enzymes of *C. thermophilum* were more abundant than those of common ascomycetes (Additional file 1: Table S2), with a total of 51 enzymes, formed by 23 hydrolases and 28 oxidases. The hydrolases were composed of 5 CBHs, 10 EGs and 8 BGs, and the oxidative enzymes included 2 CDHs, 8 glucose–methanol–choline oxidoreductases (GMC oxidoreductases) and 18 LPMOs, among which CDHs and GMC oxidoreductases can transfer electrons to LPMOs [38, 39]. Among the 18 LPMOs, which all belonged to family AA9 (Table 1), 9 LPMOs contained a separate catalytic domain, 6 AA9 LPMOs had multiple domains, 5 of which included another carbohydrate-binding module 1 (CBM1), and 3 AA9 LPMOs had regions with unknown functions. All catalytic domains of the AA9 LPMOs were located at the N-terminus of the sequence (Fig. 1a), which was consistent with the activity of AA9 LPMOs [15]. Phylogenetic tree analysis of 26 characterized AA9 LPMOs (Additional file 1: Table S3) and AA9 LPMOs in *C. thermophilum* showed that 7, 5 and 4 AA9 LPMOs belonged to LPMO1, LPMO2 and LPMO3, respectively (Fig. 1b). This result was consistent with the subfamily classification of AA9 LPMOs in the previous study [17]. Moreover, AA9 LPMOs had a flat substrate-binding surface. Compared with LPMO1, LPMO2 had a long insert in loop3. LPMO3 has an insert in loop2 (Fig. 1c), laying a foundation for further analysis of structural and functional differences among AA9 subfamilies.

In addition, the genome of *C. thermophilum* encoded 72 extracellular peptidases consisting of 37 endopeptidases and 35 exopeptidases (Additional file 1: Table S4) to efficiently degrade heterologous proteins. The numerous CAZymes and peptidase-coding genes in the genome may account for the widespread distribution of the fungus in cellulose-rich habitats.

### Dynamic changes in extracellular reducing sugars, protein concentrations and activities of GHs and LPMOs

**Fig. 2:**
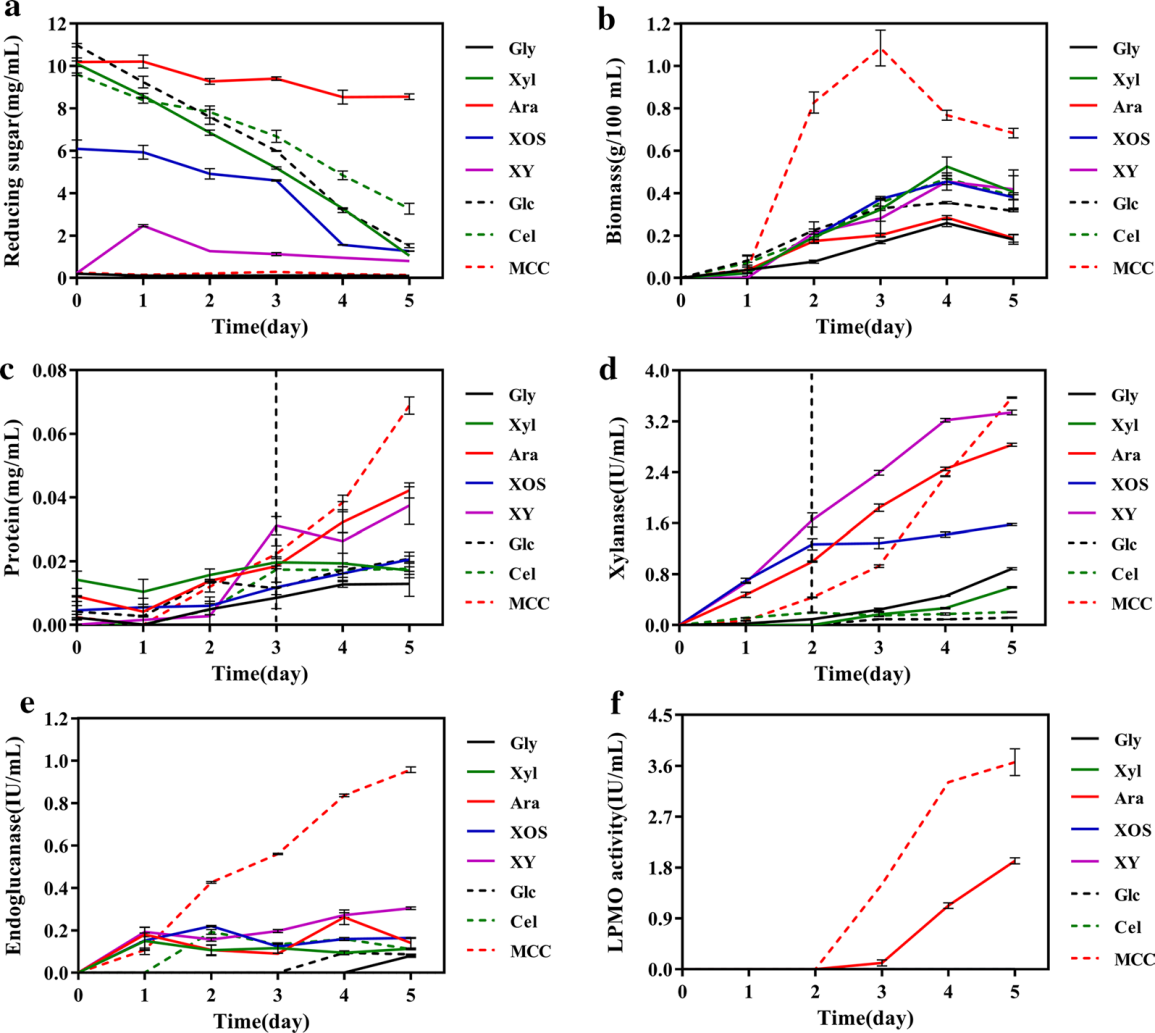
Temporal enzyme production by *C. thermophilum* during growth on different carbon sources for 5 days. **a** Reducing-sugar concentration. **b** Biomass. **c** Protein concentration. **d** Xylanase activity. **e** Endoglucanase activity. **f** LPMO activity. Bars represent standard deviations of three replicates. *Gly* glycerol, *Xyl* xylose, *Ara* arabinose, *XOS* xylo-oligosaccharide, *XY* xylan, *Glc* glucose, *Cel* cellobiose, *MCC* microcrystalline cellulose

Since the genome of *C. thermophilum* encodes a variety of cellulose- and xylan-degrading enzymes, we selected 8 cellulose- and xylan-related carbon sources for culturing *C. thermophilum* at 50 °C for 5 days. The biochemical assays were measured with the filtered culture supernatants. The concentration of extracellular reducing sugars showed that *C. thermophilum* preferentially absorbed soluble reducing sugars, such as xylose, xylo-oligosaccharide (XOS), glucose and cellobiose, since the concentration of extracellular reducing sugars decreased rapidly and the consumption reached more than 80% when cultured for 5 days. However, arabinose was an exception. The utilization rate of arabinose was only approximately 10% by *C. thermophilum*, which was different from filamentous fungi *Aspergillus niger* and *Thermomyces lanuginosus* with a utilization rate of almost 100% at day 5 [40, 41]. When *C. thermophilum* was cultured on the polysaccharide xylan, the concentration of reducing sugars increased first and then decreased, which indicated that the fungus secreted related degrading enzymes to digest polysaccharides into small sugars for rapid utilization. When *C. thermophilum* was cultured on MCC, the concentration of reducing sugars was too low to be detected (Fig. 2a).

The biomass of *C. thermophilum* increased first and then decreased among all 8 carbon sources. The difference was that the biomass of *C. thermophilum* reached the peak at day 3 and was larger than other carbon sources when cultured on MCC, while it reached the peak at day 4 when cultured on all other carbon sources (Fig. 2b). When *C. thermophilum* grew on arabinose, the biomass was lower than other soluble sugars, which was consistent with the low utilization of arabinose. The extracellular-protein content in filtered culture supernatants did not change significantly between day 1 and day 2 but increased from day 3 (Fig. 2c). Polysaccharides (xylan and MCC) induced protein secretion by *C. thermophilum* more efficiently than soluble sugars (xylose, XOS, glucose and cellobiose), except for arabinose. When cultured on arabinose, the extracellular-protein content was high, reaching 0.04 mg/mL at day 5, second only to MCC culture, although the biomass and the utilization of arabinose were low. This result indicated that arabinose could specifically induce the secretion of proteins by *C. thermophilum.* The largest extracellular-protein content was detected in MCC culture at day 5 (approximately 0.07 mg/mL) (Fig. 2c). However, the total extracellular protein secreted by *C. thermophilum* was much lower than that secreted by filamentous fungi such as *Trichoderma reesei* [42].

Although the secreted extracellular-protein content was low, glycoside hydrolase activity could be clearly detected. The xylanase activity produced by *C. thermophilum* increased markedly from day 2. The xylanase activity was higher when *C. thermophilum* was cultured on arabinose, xylan and MCC (≥2.8 IU/mL) than other carbon sources (≤ 1.6 IU/mL). The xylanase activity showed the same trend when *C. thermophilum* was cultured on arabinose and xylan, but the latter was higher, similar with the specific activity (IU/mg, Additional file 2: Fig. S1a). When cultured on MCC, the xylanase activity reached the highest level at day 5 (3.5 IU/mL) (Fig. 2d). Therefore, efficient secretion of extracellular xylanase by *C. thermophilum* should be related to arabinose and MCC and was different from that of filamentous fungi *A. niger* and *T. lanuginosus* [40, 41], which their efficient secretions of extracellular xylanase were related to xylose. When cultured on MCC, the endoglucanase activity increased rapidly from day 2, reaching 1 IU/mL at day 5 but was lower than 0.2 IU/mL when cultured on other carbon sources (Fig. 2e). Similarly, the specific activity of endoglucanase (IU/mg) was much higher when *C. thermophilum* was cultured on MCC than that of other carbon sources (Additional file 2: Fig. S1b). Therefore, MCC could specifically induce the cellulose-degrading enzymes of *C. thermophilum*.

In addition to glycoside hydrolase activities, the LPMO activity of extracellular protein was only detected in filtered culture supernatants when *C. thermophilum* was cultured on arabinose and MCC, and the activity when *C. thermophilum* was cultured on the latter was higher (Fig. 2f and Additional file 2: Fig. S1c). The activity of LPMOs was detected at the later stage of growth (between days 3 and 5). As a result, arabinose and MCC could specifically induce the secretion of LPMOs by *C. thermophilum*.

### Comparative analysis of functional secretomes of *C. thermophilum* when cultured on different carbon sources

The secretomes of *C. thermophilum* growing for 5 days on different carbon sources were analyzed by LC–MS/MS. The secretion of CAZymes by *C. thermophilum* was substrate-dependent. Among secretomes, 96±4, 90±8, 90±9 and 82±4 species of CAZymes could be detected in secretomes induced by xylan, MCC, arabinose and glycerol, respectively. Glycerol was the last one among these four carbon sources for it was just as the non-inducing carbon source [43], and it did not have the carbon catabolite repression for *C. thermophilum* compared with glucose, cellobiose and xylose [44–46]. Xylan and MCC are polysaccharides with complex structures, so more CAZymes had been secreted by *C. thermophilum* to utilize the substrate. Although arabinose was a soluble sugar, but it could induce more species of CAZymes, which was different from other soluble sugars, so arabinose was a good inducer for *C. thermophilum*. In addition, the secretome induced by arabinose and MCC contained the most species and the highest relative content of AAs (Additional file 2: Fig. S2a and b).

**Fig. 3:**
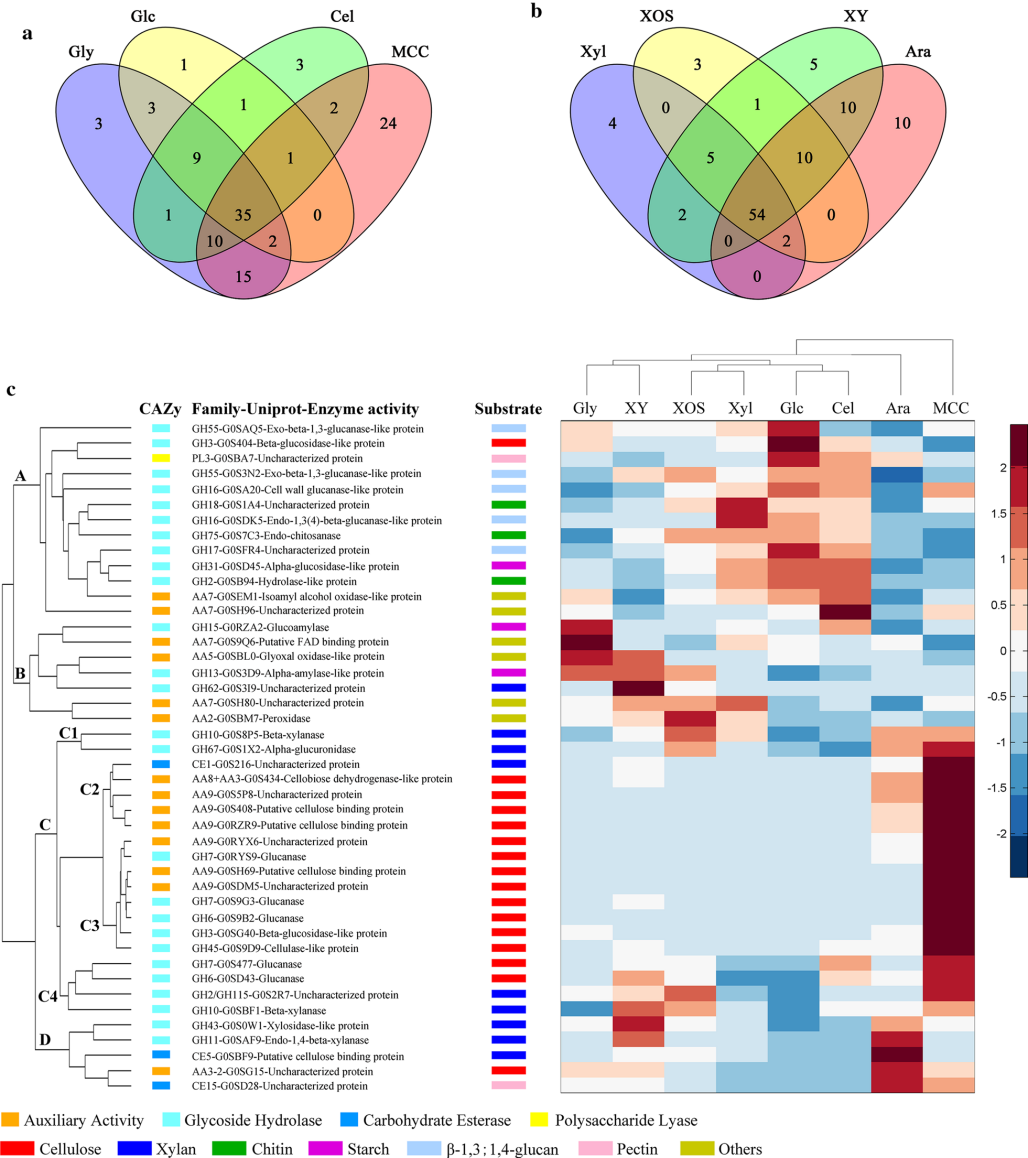
Venn diagram and dicluster comparing related CAZymes from secretomes of different carbon sources. **a** Venn diagram of CAZymes from glycerol and cellulose-related carbon sources (Glc, Cel and MCC). **b** Venn diagram of CAZymes from xylan-related carbon sources (Xyl, XOS, XY and Ara). **c** Dicluster of selected CAZymes detected in secretomes of *C. thermophilum* induced by different carbon sources. The color intensity is related to the relative content of each enzyme

CAZymes related to the degradation of plant biomass and chitin were selected from all detected proteins (Additional file 1: Table S5). The results showed that the species and the relative content of enzymes that degrade pectin, galactomannan and xyloglucan were very low, which were consistent with the fact that there were few genes encoding related enzymes in the genome (Additional file 1: Table S1), suggesting that *C. thermophilum* could not degrade dicots efficiently. To provide better insight into the pattern of related CAZymes in different carbon sources, these CAZymes were mapped in the Venn diagrams. The result showed that there were 24 MCC-specific CAZymes among secretomes of glycerol and cellulose-related carbon sources (glucose, cellobiose and MCC) (Fig. 3a), in accordance with their functions in Additional file 1: Table S6; in Fig. 3b, there were 10 arabinose-specific CAZymes among secretomes of 4 xylan-related carbon sources (xylose, XOS, xylan and arabinose) (see Additional file 1: Table S7 for details). MCC and arabinose were the only two carbon sources that induced the carbon-specific secretion of AA9 LPMOs (Additional file 1: Table S6 and S7).

Comparative analysis of CAZymes species using Venn diagrams did not consider the relative contents of CAZymes, so the most abundant CAZymes with the relative content greater than 0.5% in each secretome were ordered in Additional file 1: Table S8 and clustered (Fig. 3c). Most of them had N-terminal secretion signals, and the secretome of MCC had the most species of CAZymes (26 species) with a relative content greater than 0.5%, while the species of others were between 11 and 15. The clustering results showed that branches A and B represented CAZymes that were induced by almost all 8 carbon sources, in accordance with most CAZymes shared among all carbon sources (Fig. 3a, b), although the relative contents of CAZymes in branch A were more abundant in secretomes of glucose, cellobiose and xylose, and secretomes of glycerol, XY and XOS had more relative contents of CAZymes in branch B. In particular, branch C contained 14 and 5 CAZymes that involved in cellulose and xylan degradation, respectively. CAZymes in branch C had an especially pronounced induction in the presence of MCC and a partial induction in the presence of arabinose (branch C1 and C2). The branch D represented CAZymes induced by most carbon sources but especially by the presence of arabinose. The CAZymes expression patterns of arabinose and MCC seemed distinguishable from others, which were independently branched while others were clustered together vertically (Fig. 3c).

Arabinose could induce the secretion of CAZymes related to xylan degradation, with a total relative content of 8%, mainly including G0SBF9 (CE5), an endo-β-1,4-xylanase (XYN) G0SAF9 (GH11), a β-xylosidase-like protein G0S0W1 (GH43) in branch D and G0S8P5 (XYN, GH10), an α-glucuronidase (AGU) G0S1X2 (GH67) in branch C1. In addition to xylanases, arabinose also induced the secretion of some oxidative enzymes (mainly in branch C2) related to cellulose degradation by *C. thermophilum*, with a total relative content of 9% (Fig. 3c and Additional file 2: Fig. S2c). Among them, 9 AA9 LPMOs were detected with a total relative content of 4.2%. When cultured on MCC, *C. thermophilum* specifically secreted a large number of cellulose-degrading enzymes (mainly in branch C, Fig. 3c), with a relative content of about 41%. The relative contents of hydrolases and oxidases were about 22% and 19%, respectively (Additional file 2: Fig S2b). Among the oxidases, twelve AA9 LPMOs were detected with a total relative content of 13.8%. The results also showed that MCC could specifically induce the secretion of AA9 LPMOs by *C. thermophilum* with the most species and the highest content, followed by arabinose. AA9 LPMOs were hardly induced by other carbon sources, which were consistent with the enzyme activity (Fig. 2f).

Compared with other carbon sources, the relative content of xylan-degrading enzymes was higher when *C. thermophilum* was cultured on xylan, XOS, arabinose and MCC (Additional file 2: Fig. S2c), except for xylose. The relative contents were consistent with the activities of xylanase (correlation coefficient R = 0.8, Fig. S2d), indicating that arabinose and MCC were efficient inducers of xylanase secretion by *C. thermophilum*. In addition, MCC also induced the highest content of endoglucanases (approximately 4%), which was consistent with endoglucanase activity (R = 0.98, Additional file 2: Fig. S2d). Therefore, both cellulases and xylanases could be induced efficiently by MCC.

### Temporal changes in secretomes induced by arabinose and MCC

As arabinose and MCC could specifically induce the secretion of AA9 LPMOs by *C. thermophilum*, we further analyzed temporal changes (days 1, 3 and 5) in the secretomes of *C. thermophilum* by LC–MS/MS. The results showed that the total species of extracellular CAZymes induced by both carbon sources increased first and then decreased (Additional file 2: Fig. S3a). When *C. thermophilum* was cultured on MCC, the relative content of CAZymes increased over time; the relative content of GHs remained unchanged, but the relative content of AAs increased (Additional file 2: Fig. S3b).

**Fig. 4:**
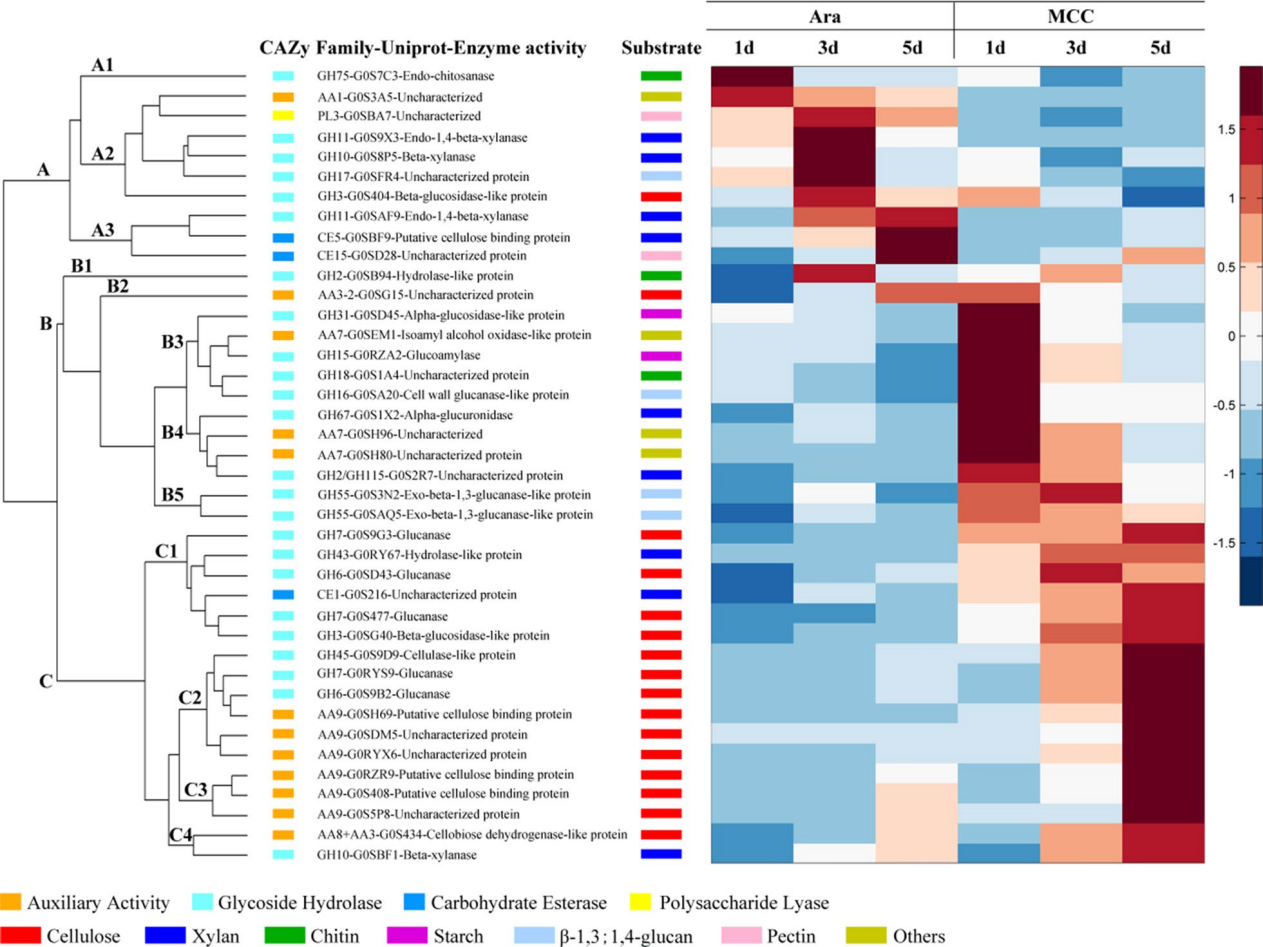
Heat map comparison of expression patterns of selected CAZymes detected in the secretomes of *C. thermophilum* during growth on arabinose and MCC at days 1, 3, and 5. The color intensity is related to the relative content of each enzyme

We further selected the CAZymes participating in the degradation of plant biomass and chitin from all detected proteins (Additional file 1: Table S9), and enzymes with a relative content greater than 0.5% were clustered. The results showed that extracellular enzymes secreted by the fungus had an obvious change with time when cultured on arabinose and MCC (Fig. 4). When induced by arabinose, *C. thermophilum* mainly secreted xylanases. The XYNs G0S9X3 and G0S8P5 belonged to families GH11 and GH10, respectively, and their relative contents increased first and decreased rapidly in cluster A2 (Fig. 4). In addition, the relative contents of XYN of GH11 (G0SAF9) and CE of CE5 (G0SBF9) increased in cluster A3 (Fig. 4). Moreover, the relative contents of XYN of GH10 (G0SBF1) and some oxidases increased rapidly at day 5 in clusters C4 and C3 (Fig. 4). The relative content of AA9 LPMOs in oxidases was 4.24%, second to XYNs (Additional file 2: Fig. S3c and d).

When cultured on MCC, 2 starch-degrading enzymes, 2 chitin-degrading enzymes, 3 β-1,3;1,4-glucanases and 2 AGUs were secreted by *C. thermophilum* in the early stage (day 1) and decreased with time (Fig. 4). In addition, the aggregation of various cellulose-degrading enzymes and some xylanases in cluster C showed an increasing trend with time (Fig. 4). This indicated the specialization and coexpression of these enzymes, suggesting that their coding genes may share transcriptional regulators or mechanisms, similar to the CLR-1 and CLR-2 in the network of plant cell wall deconstruction by *Neurospora crassa* [47–49]. These xylanases included an arabinofuranosidase (ABF)-like of GH43 (G0RY67), a feruloyl esterase of CE1 (G0S216) and an XYN of GH10 (G0SBF1). G0SBF1 is a special xylanase with CBM1. A study showed that the CBM1 of PspXyn10 could bind to cellulose [50]. Therefore, it was hypothesized that the CBM1 of G0SBF1 could bind to the surface of cellulose. Moreover, a large number of cellulose-degrading enzymes, including hydrolases and oxidases, were secreted with time, and the total relative content of cellulose-degrading enzymes increased rapidly from 16% (day 1) to 41% (day 5) (Additional file 2: Fig. S3c). The proportion of CBH accounted for 69% of all cellulose hydrolases at day 5. Cellulose oxidases contain LPMOs, CDHs and GMC oxidoreductases. The content of LPMOs accounted for the majority of cellulose oxidases and increased rapidly from 4.2% (day 1) to 13.8% (day 5) (Additional file 2: Fig. S3d). CDHs and GMC oxidoreductases could act as electron donors for LPMOs with a low proportion. In addition, the relative contents of LPMOs were consistent with 2,6-DMP activity measured previously (R=0.96, Additional file 2: Fig. S3f).

The results showed that 6 AA9 LPMOs, including 2 LPMO1s, 1 LPMO3 and 3 LPMO2s, were highly secreted by *C. thermophilum* when cultured with MCC. Two LPMO1s (G0RZR9 and G0S408) and 1 LPMO3 (G0S5P8) were clustered together in cluster C3 (Fig. 4). They were similar to *Pa*LPMO9E (sequence identity 62.3%), *Mt*LPMO9B (76.1%) and *Pa*LPMO9A (63.5%), respectively, which only acted on cellulose substrates based on published studies [22, 51]. These LPMOs all had a CBM1, which could strengthen the binding capacity of enzyme to cellulose [52], and could be induced by both arabinose and MCC. Interestingly, 3 LPMO2s (G0SH69, G0SDM5 and G0RYX6) were clustered in cluster C2 and induced specifically by MCC (Fig. 4); they were similar to *Mt*LPMO9C (85.7%), NCU02916 (61.6%) and *Pa*LPMO9H (75.5%), respectively [22, 24, 51]. Among them, G0SH69 and G0SDM5 only had catalytic domains, while G0RYX6 had another CBM1.

*Chaetomium thermophilum* secreted more LPMO1s and LPMO2s when cultured on MCC than when cultured on arabinose (Additional file 2: Fig. S3e). The correlation analysis between LPMOs and cellulose hydrolases, CDHs and GMC oxidoreductases showed that LPMO2s had a higher correlation with CBH (R=0.93) and CDH (R=0.87), compared with LPMO1s (Additional file 2: Fig. S4). However, LPMO2s had a lower correlation with GMC oxidoreductases (Additional file 2: Fig. S4). Therefore, CDH may be the main electron donor in the extracellular system for LPMOs. This result suggested that LPMO2, CBH and CDH were efficiently cosecreted, thus forming an efficient cellulose degradation system of oxidative and hydrolytic enzymes.

### Quantitative transcript analysis of key functional enzymes under arabinose and MCC induction

**Fig. 5:**
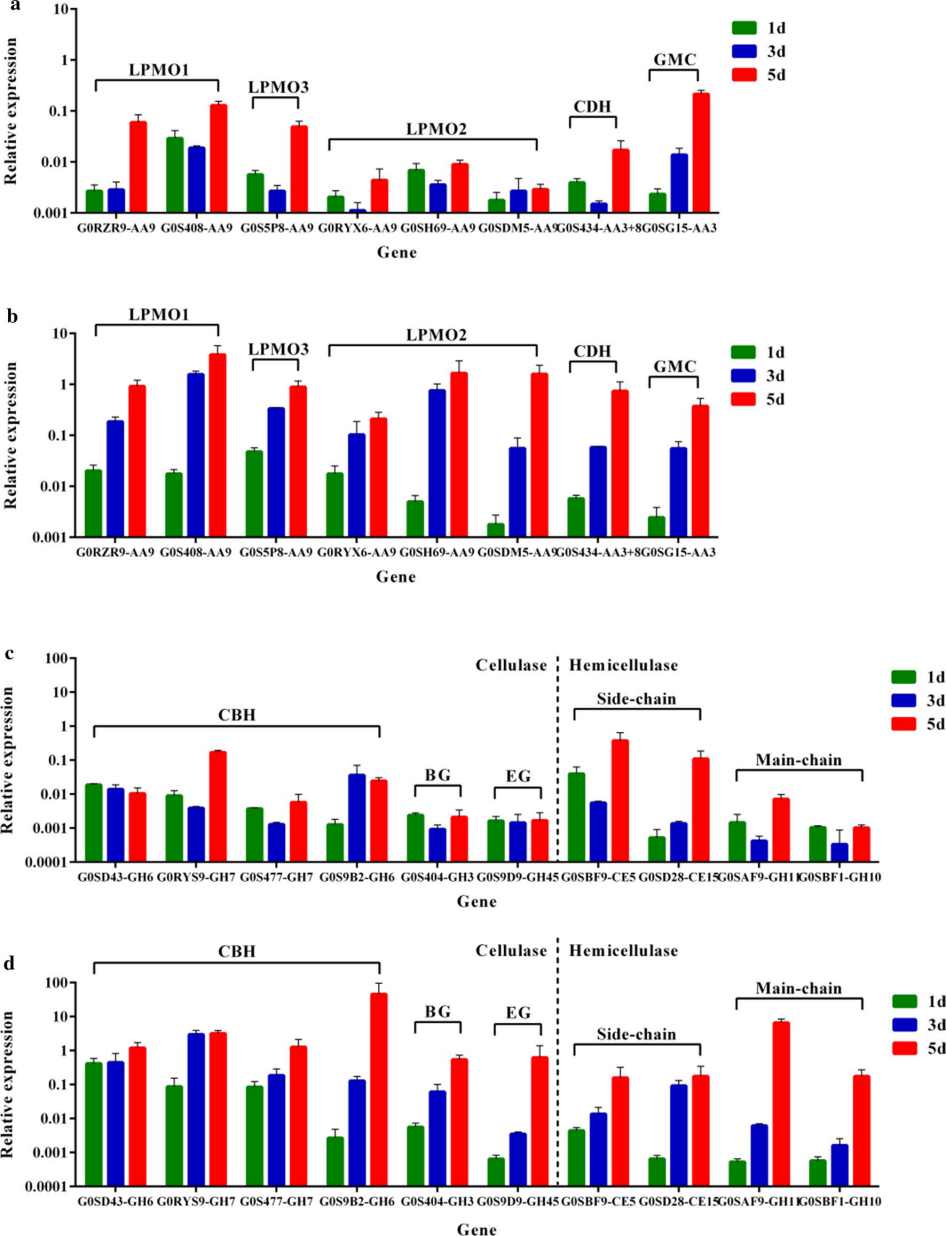
Expression levels of selected enzyme-coding genes determined using RT-qPCR. **a** Selected oxidase-coding genes (LPMO3, G0S5P8-AA9; LPMO1, G0RZR9-AA9, G0S408-AA9; LPMO2, G0RYX6-AA9, G0SH69-AA9, G0SDM5-AA9; CDH, G0S434-AA3+AA8; GMC, G0SG15-AA3) induced by arabinose. **b** Selected oxidase-coding genes induced by MCC. **c** Selected genes related to cellulose and hemicellulose degradation (CBH, G0SD43-GH6, G0RYS9-GH7, G0S477-GH7 and G0S9B2-GH6; BG, G0S404-GH3; EG, G0S9D9-GH45; hemicellulose side-chain, G0SBF9-CE5 and G0SD28-CE15; main-chain, G0SAF9-GH11 and G0SBF1-GH10) induced by arabinose. **d** Selected genes related to cellulose and hemicellulose degradation induced by MCC

Proteome analysis can accurately analyze changes in extracellular enzyme species, and quantitative real-time PCR (RT-qPCR) can accurately quantify the transcript levels of related genes. Therefore we chose 18 genes encoding enzymes that were highly expressed in MCC culture, including 8 oxidases (6 LPMOs, 1 CDH and 1 GMC oxidoreductase), 6 cellulose hydrolases (4 CBHs, 1 BG and 1 EG) and 4 hemicellulases (2 XYNs and 2 CEs) (Fig. 5). The expression patterns of these genes relative to that of 3-p-glyceraldehyde dehydrogenase at different times (days 1, 3 and 5) in *C. thermophilum* grown on arabinose and MCC were investigated (Fig. 5).

The results showed that the transcript levels of most genes were obviously higher with MCC induction compared with arabinose induction, except for G0SBF9 of CE5, and showed an uptrend with time (Fig. 5). When induced by MCC at day 5, the transcript levels of 2 LPMO1s (G0RZR9 and G0S408) and 1 LPMO3 (G0S5P8) were 14.4-, 28.7- and 17.1-fold higher than those induced by arabinose, respectively. In addition, the transcript levels of 3 LPMO2s (G0RYX6, G0SDM5 and G0SH69) were hardly induced by arabinose (Fig. 5a) but significantly increased with time under MCC induction (Fig. 5b), which was in good agreement with the relative content of proteins identified by LC–MS. The results of gene transcription and secretome analysis suggested that AA9 LPMO2s could be specifically induced by MCC. The transcript level of CDH-G0S434 under MCC induction was 42-fold higher than that under arabinose induction at day 5, while the transcript level of GMC-G0SG15 was similar in both conditions. In addition to oxidases, the transcript levels of cellulose hydrolases were low under arabinose induction (Fig. 5c). However, the transcript levels of these enzymes were high with MCC induction (Fig. 5d). The correlation analysis between the transcript levels of LPMO subfamily and corresponding enzymes showed that the transcript level of LPMO2 had a higher correlation with CBH, BG and CDH, compared with LPMO1 (Additional file 2: Fig. S5). Combined with the results of secretomes, we could suggest that LPMO2, CBH and CDH were efficiently coexpressed.

CE5-G0SBF9, a gene that encodes xylan side-chain enzyme, was the only gene that had a higher transcript level under arabinose induction compared with MCC induction. The transcript level of GH10-G0SBF1 was 170.7-fold higher at day 5 under MCC induction compared with arabinose induction. The transcript levels of these genes were in good agreement with the proteomic analysis of secretomes. Through gene transcription analysis, it was suggested that GH10-G0SBF1 was coexpressed with most cellulose-degrading enzymes.

## Discussion

**Fig. 6:**
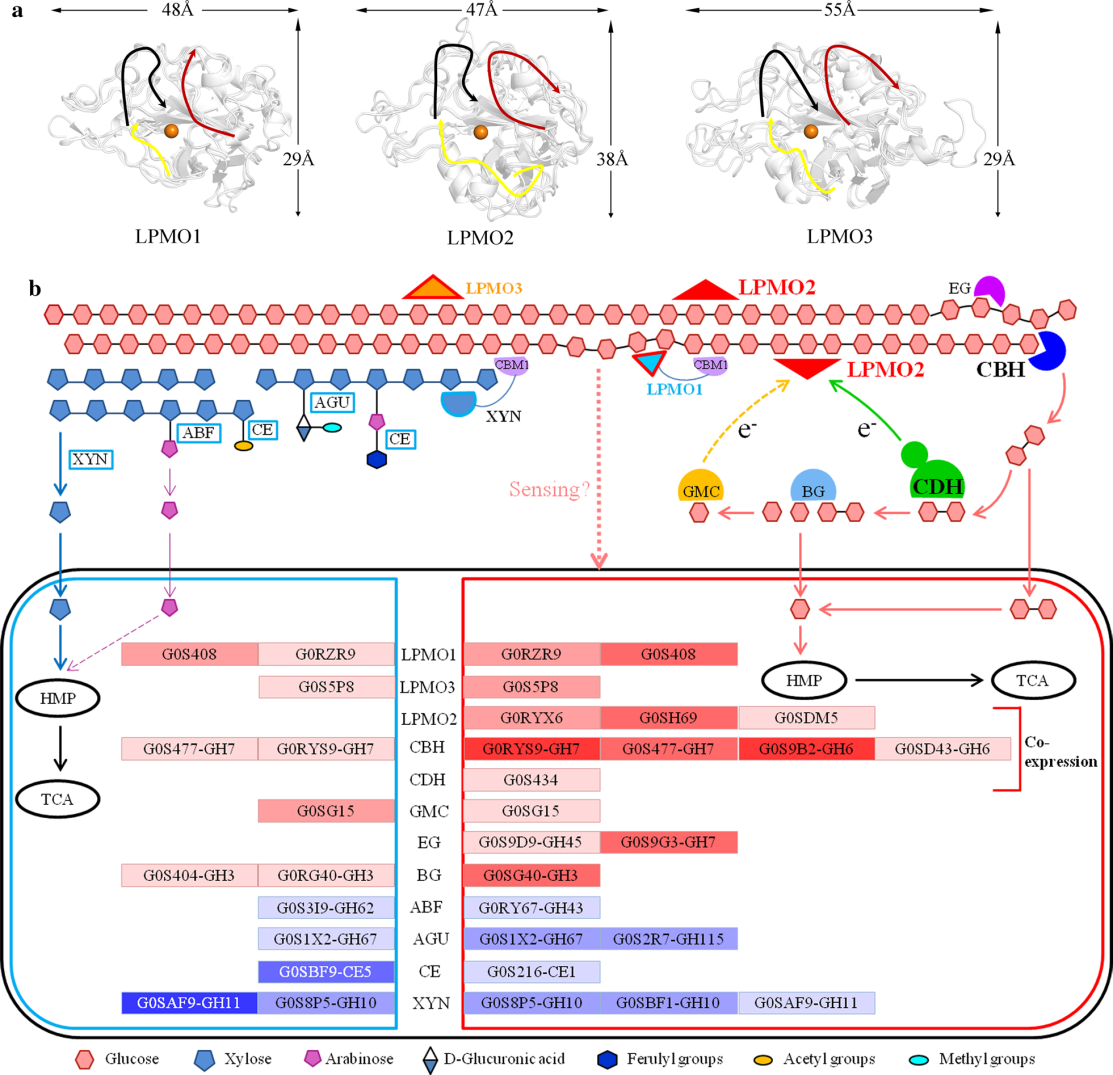
**a** Substrate-binding surface in the modeled structures of the AA9 subfamily in *C. thermophilum*. Three regions (depicted by black, red and yellow arrows) define the immediate environment of the catalytic center. **b** Schematic diagram representing the synergistic lignocellulose degradation mode of *C. thermophilum*. Solid boxes indicate the enzyme genes that were detected in the secretomes of *C. thermophilum*. A greater saturation of the color of the box indicates more expression of the proteins, and vice versa. Blue and purple arrows indicate the degradation of xylan, and red arrows indicate the degradation of cellulose; the purple dotted line represents that arabinose hardly enters the HMP pathway. The green solid arrow indicates direct electron transfer, and the yellow dotted arrow indicates indirect electron transfer

Plant biomass is recognized as a sustainable source of mixed sugars for fermentation to second generation biofuels and high-value molecules. Fungi have evolved different strategies to degrade lignocellulose [53–58]. The genome of the thermophilic fungus *C. thermophilum* suggested that this fungus should prefer to degrade monocots that are rich in cellulose, xylan and β-glucan. CAZymes in the functional secretomes of *C. thermophilum* were substrate-dependent, and the soluble sugar arabinose and insoluble substrate MCC were specific for CAZyme induction (Fig. 6). Xylanase activity, protein concentration and functional secretome analysis indicated that arabinose was a good inducer of xylanases secreted by *C. thermophilum*, which was different from filamentous fungi such as *A. niger* and *T. lanuginosus* [40, 41]. *C. thermophilum* could secrete enzymes that hydrolyze the side chains of xylan to produce arabinose, but the absorption of arabinose by the fungus was weak because *C. thermophilum* lacks arabinose reductases based on the analysis of its genome, which was mapped to KEGG pathways (Additional file 2: Fig. S6). Therefore, arabinose generated by degradation may be further absorbed by other microorganisms. *T. lanuginosus* could absorb arabinose well for growth and degrade xylan efficiently but could not utilize cellulose [41, 59, 60] while *C. thermophilum* could degrade cellulose well, showing a partial complementary in their ability to degrade lignocellulose. What’s more, a study had shown that the thermophilic fungi *C. thermophilum* and *T. lanuginosus* coexisted in the same habitats, which had relatively high temperatures and lignocellulose-rich composts [61]. So we suppose that *C. thermophilum* may be symbiotic with *T. lanuginosus*, which needs more effort for further study.

The insoluble crystalline substrate MCC specifically induced the efficient secretion of cellulose-degrading enzymes by *C. thermophilum*, which was similar to the model fungus *N. crassa* [62]. However, *N. crassa* is a mesophilic fungus with an optimum growth temperature of 30 °C, and its cellulose-degrading enzymes encoded by the genome are similar to those of *C. thermophilum* (Additional file 1: Table S2). The difference is that *C. thermophilum* is a thermophilic fungus whose genome encodes more CBHs and LPMOs. Gene expansion probably reflects the adaptability of the fungus to the substrate [29]. A study that analyzed the genomes and proteomes of thermophilic fungi and mesophilic filamentous fungi found the common strategies of thermal adaptation, including amino acid biases and a reduced genome size. Consistent amino acid substitutions associated to thermophily, especially substitution of lysine by arginine, are common in thermophilic fungi. Moreover, the loop rigidity by increased praline frequency, increase protein core hydrophobicity, and the increased electrostatic interactions stabilizing neighboring secondary structure elements are also the different types of the adaptive mutations in thermophilic fungi. Finally, *C. thermophilum* are rich in cysteines that might contribute to thermophily in several ways [63]. These strategies probably explain why CAZymes of thermophilic fungi are thermal stable under thermophilic conditions.

The temporal secretomes and transcriptional analysis of *C. thermophilum* indicated that G0SBF1, a xylanase of GH10 with CBM1, was coexpressed with most cellulose-degrading enzymes. It was suggested that CBM1 binds to the surface of cellulose, and then G0SBF1 degrades the residual xylan with sidechains attached to the outermost layer of cellulose, thus removing obstacles for cellulases to degrade cellulose. MCC could specifically induce the secretion of 3 LPMO2s, suggesting that LPMO2s may be the main LPMOs that efficiently degrade crystalline cellulose under thermophilic conditions. LPMO2s, CBHs and CDHs could be efficiently coexpressed under MCC induction and form an efficient cellulose-degradation system of oxidative and hydrolytic enzymes under thermophilic conditions. In addition to LPMO2s, the fungus also secreted LPMO1s and LPMO3s under arabinose and MCC induction.

Compared with LPMO1s and LPMO3s, the substrate-binding surface of LPMO2 has an insert in loop3. On the one hand, this insert contains more polar amino acids (E, D, N, K, S) and aromatic amino acids (W, Y, H); on the other hand, this insert also results in a visible extension of the substrate-binding surface (Fig. 6a), contributing to the binding of enzymes to the region of crystalline cellulose [64]. Therefore, LPMO2s bind to crystalline cellulose and cleave the chain, thus providing more adhesion sites for CBH. CBH degrades the cellulose chain to produce cellobiose, and CDH dehydrogenates cellobiose and transfers a molecular electron to LPMOs under thermophilic conditions (Fig. 6b). This coexpression enzyme system may contribute to the fast degradation of cellulose and the wide distribution of *C. thermophilum* in cellulose-rich and self-heating habitats.

LPMOs can produce H_2_O_2_, a byproduct of the oxidation reaction, in the absence of substrates [65]. These oxidative free radicals are toxic to microbial cells. In addition to cellulases and xylanases, the genome of this fungus also encodes many β-1,3-glucan and chitin-degrading enzymes, which are favorable for the degradation of fungal cell walls. This may also be one of the reasons why *C. thermophilum* is the dominant microorganism in self-heating composts [66]. Although *C. thermophilum* secretes a low concentration of extracellular protein under MCC induction, the extracellular enzymes form an efficient cellulose-degradation system under thermophilic conditions. Thermophilic enzymes also have many advantages in practice; therefore, further optimization to improve protein secretion is of great significance for industrial applications. Thermophilic enzymes can also be heterogeneously expressed to improve the protein concentration, laying a foundation for further design of optimal enzyme cocktails for more efficient exploration of biomass resources under thermophilic conditions.

## Conclusions

*Chaetomium thermophilum* is a thermophilic fungus widely distributed in lignocellulosic-degrading habitats. Its genome encodes a comprehensive range of cellulose- and xylan-degrading enzymes, especially 18 AA9 LPMOs, belonging to different subfamilies such as LPMO1, LPMO2 and LPMO3. Extracellular secretomes and transcription analysis showed that out of 8 carbon sources, arabinose and MCC were specific for the induction of extracellular CAZymes. Arabinose induced the secretion of xylanases by *C. thermophilum*, which is obviously different from other common filamentous fungi. MCC could efficiently induce the special secretion of LPMO2s, possibly because the insert in loop3 on the substrate-binding surface of LPMO2s strengthens their binding capacity to cellulose. LPMO2s, CBHs and CDHs were cosecreted, forming an efficient cellulose degradation system of oxidases and hydrolases under thermophilic conditions. This novel insight increases our understanding of the degradation of cellulose and may inspire the design of optimal enzyme cocktails for more efficient exploration of biomass resources under thermophilic conditions.

## Materials and methods

### Bioinformatics analysis of the genome of *C. thermophilum*

The genome information of *C. thermophilum* DSM 1495 was obtained from published studies [33, 67] and the NCBI database (https://www.ncbi.nlm.nih.gov/). Sequences of AA9 LPMOs used in this study included 18 AA9 LPMOs in the genome of *C. thermophilum* and 26 AA9 LPMOs characterized in the CAZy database (http://www.cazy.org/). First, the domain composition of the 18 AA9 LPMOs was analyzed according to their sequences, and then models of their domain compositions were drawn in IBS-Database Visualization (http://ibs.biocuckoo.org/dbvisualization.php).

To construct phylogenetic trees, any noncatalysis domains, such as CBM and linkers, were deleted. Multiple sequence alignment was performed with Clustal W (gap open = 10.0, gap extend = 0.2) and the phylogenetic tree was drawn with MEGA and optimized in iTOL (https://itol.embl.de/itol.cgi).

For AA9 LPMOs in *C. thermophilum*, the Swiss Model (https://swissmodel.expasy.org/) was used to build suitable models. Amino acids on substrate-binding surface of the structure were selected by Pymol and then recorded. Based on the results of multiple sequence alignment, we obtained the substrate-binding site architecture of the whole family.

### Fungal strain and cultivation conditions

The filamentous fungus used in this work was the thermophilic fungus *Chaetomium thermophilum* CGMCC3.17990, provided by Doctor Duochuan Li of Shandong Agricultural University. Glycerol, glucose, cellobiose, MCC, xylose, arabinose, XOS and xylan were purchased from Sangon Biotech Co., Ltd. (Shanghai, China) and used as different carbon sources. Spores of the strain were kept in 80% (v/v) glycerol at − 20 °C. One percent (w/v) of the different carbon sources was added to minimal medium (MM) as described previously [40]. All media were sterilized at 115 °C for 30 min. Two hundred microliters of spores (7 × 10^7^/mL) were inoculated into 100 mL of medium and cultured at 50 °C with shaking at 200 rpm for 5 days, and samples were collected every day. Substrates and fungal biomass were removed using eight layers of gauze and centrifuged at 8000 rpm at 4 °C for 15 min. The supernatants were stored at 4 °C and used in further experiments.

### Extracellular reducing sugars, protein and enzyme activity assays

The dry weight of fungal mycelium was used to characterize the total biomass [68]. Gauze was used to filter the mycelium, and they were dried together at 50 °C until a constant weight was obtained. The Bradford method was used to determine the protein concentrations in the filtered culture supernatants: 100 μL of protein sample and 1 mL of Coomassie Brilliant Blue G-250 dye were reacted at room temperature for 10 min. Each sample was tested in triplicate, and all mixtures were measured at 595 nm using a microplate spectrophotometer (Tecan, Morrisville, NC, USA). Bovine serum albumin (0.1 mg/mL) was used to obtain the standard curve.

The concentrations of reducing sugars and endoglucanase and xylanase activity were measured using the dinitrosalicylic acid method. One percent xylan (w/v) or 1% sodium carboxymethylcellulose (CMC, Sangon Biotech, Shanghai, China) (w/v) in sodium hydrogen phosphate/citric acid buffer (pH 6.0) was used as a substrate to measure xylanase or endoglucanase activities. The filtered culture supernatant (400 μL) and 600 μL of 1% xylan were mixed and reacted at 60 °C for 30 min to measure the xylanase activity; The filtered culture supernatant (400 μL) and 600 μL of 1% CMC were mixed and reacted at 70 °C for 30 min to measure the endoglucanase activity. Then 800 μL of DNS was added to each sample and boiled for 10 min. The mixture was made up to 10 mL, and the absorbance at 550 nm was measured with an ultraviolet spectrophotometer (Puyuan Instruments, Ltd., Shanghai, China). The standard curve was prepared using 1 mg/mL xylose or glucose.

LPMO activity was determined by the 2,6-DMP method [69]. A total of 780 μL of 128 mM succinic acid/phosphate buffer (pH 6.0) was added to 100 μL of 10 mM 2,6-DMP and 20 μL of 5 mM H_2_O_2_. After mixing, the mixture was added to a cuvette and incubated at 50 °C for 15 min, and 100 μL of protein sample was added and mixed. Then, the increase in absorbance was measured at 469 nm wavelength for 300 s.

### Analysis of proteins by liquid chromatography tandem mass spectrometry (LC–MS/MS)

The filtered culture supernatant was ultrafiltered using a 3-kDa cutoff membrane and then precipitated using 10% (w/v) trichloroacetic acid (TCA, Sigma-Aldrich) and 0.1% dithiothreitol (DTT, Sigma-Aldrich) dissolved in acetone. Then, the precipitated protein was dried and dissolved in ultrapure water. After determining the protein concentration by using the Bradford method, 50 μg of protein solution was mixed with 50 μL of degeneration buffer (0.5 M Tris–HCl, 2.75 mM EDTA and 6 M guanidine–HCl; Sigma-Aldrich) and 30 μL of 1 M DTT and then incubated at 37 °C for 2 h. Fifty microliters of 1 M iodoacetamide (IA, Sigma-Aldrich) was added, and the mixture was incubated in the dark for 1 h for alkylation. The mixture was transferred to a Microcon YM-10 centrifuge tube (3-kDa membrane; Millipore) and then washed three times with 360 μL of NH_4_HCO_3_ (25 mM; Sigma-Aldrich) by centrifugation at 14,000×*g* for 15 min at 4 °C. The washed protein was obtained by centrifugation at 1000×*g* for 15 min at 4 °C and digested with trypsin at a ratio of 1:25 (w/w) at 37 °C overnight. After desalination through a C18 ZipTip (Millipore, Burlington, MA, USA), the peptide sample was dissolved with 0.1% (v/v) trifluoroacetic acid (TFA, Sigma-Aldrich) and subjected to LC–MS/MS analysis on a Prominence nano LC system (Shimadzu, Kyoto, Japan) coupled with an LTQ-Orbitrap Velos Pro ETD mass spectrometer (Thermo Fisher Scientific, Waltham, MA, USA). A custom-made silica column (75 μm × 15 cm) packed with Reprosil-Pur 120 C18-AQ (Dr. Maish GmbH, Ammerbuch, Germany) was used to separate peptides, which were eluted with a stepping gradient of solvent A (2.0% ACN in water [v/v] with 0.1% [v/v] formic acid) and solvent B (98% ACN in water [v/v] with 0.1% [v/v] formic acid). A nanospray ion source with a voltage of 2000 V and a transfer capillary temperature of 275 °C was used to spray the separated peptides into the mass spectrometer. The system was run in data-dependent acquisition mode using Xcalibur 2.2.0 software (Thermo Fisher Scientific) to perform MS/MS experiments. Full scan MS spectra (from 400 to 1800 m/z) were detected in the Orbitrap with a resolution of 60,000 at 400 m/z. The ten most intense precursor ions greater than the threshold of 5000 counts in the linear ion trap were selected for MS/MS fragmentation analysis at a normalized collision energy of 35%. Two replicates were performed for each sample that cultured on different carbon sources. Three replicates were performed for each sample that cultured on arabinose and MCC at different times.

### Database searches

Proteome Discover software version 1.4 (Thermo Fisher Scientific) with the SEQUEST search engine was used for data searches. The reference database of *C. thermophilum* was downloaded from UniProt (http://www.uniprot.org). Parameters for MS/MS searches were set as follows: (1) trypsin was used to digest the proteins, allowing two missed cleavages; (2) precursor mass tolerance was set at 10 ppm, with a fragment mass tolerance of 0.8 Da; and (3) oxidation of methionine was chosen as the dynamic modification, as well as carbamidomethylation of cysteine residues for the fixed modification. Only peptides with at least six amino acid residues showing 95% certainty (*q* ≤ 0.05) were included in the results, and the false discovery rate was set at 1%. The relative abundance of proteins was characterized by peptide spectrum matches (PSMs). Previous studies have demonstrated that there was a linear correlation between PSMs and protein abundance [70]. The SignalP 4.1 Server (http://www.cbs.dtu.dk/services/SignalP/) was used to predict the secretion signals of proteins. Venn diagram of protein species was done using Venny 2.1 (https://bioinfogp.cnb.csic.es/tools/venny/index.html). A relative content of each protein is the ratio of the PSM value of this protein to the PSM value of total proteins. Post-processing was done using Matlab R2014a, hierarchical clustering and heat map generation were done with Euclidean distance metric and average linkage.

### Expression analyses (RT-qPCR)

The mycelium of *C. thermophilum* was collected after 1, 3, and 5 days of growth in arabinose and MCC medium. Total RNA was extracted using the TRIzol reagent (TaKaRa, Japan) and cDNA synthesis was performed using the Evo M-MLV RT kit with gDNA clean for qPCR (AG, China). Primers of selected genes were used to amplify the corresponding genes. RT-qPCR was performed on qTOWER^3^G (Jena, Germany) using SYBR^®^ Green Pro Taq HS (AG, China). Three biological replicates and two experimental replicates were required for one sample. The GAPDH gene was used for data normalization. The primers used in the RT-qPCR analysis are listed in Additional file 1: Table S10.

## Supplementary information

**Additional file 1: Supplementary Tables (S1–S10).**

**Additional file 2: Supplementary Figures (S1–S6).**

## Data Availability

All data supporting the conclusions of this article are included with the manuscript and in the additional information.

## Abbreviations

LPMO: Lytic polysaccharide monooxygenase
AA: Auxiliary activity enzyme
MCC: Microcrystalline cellulose
CBH: Cellobiohydrolase
CDH: Cellobiose dehydrogenase
GH: Glycoside hydrolase
EG: Endoglucanase
BG: β-Glucosidase
CAZyme: Carbohydrate-active enzyme
CE: Carbohydrate esterase
PL: Polysaccharide lyase
GT: Glycoside transferase
GMC: Glucose–methanol–choline
CBM: Carbohydrate-binding module
XOS: Xylo-oligosaccharide
LC–MS/MS: Liquid chromatography–tandem mass spectroscopy
XYN: Endo-β-1,4-xylanase
AGU: α-Glucuronidase
ABF: Arabinofuranosidase
RT-qPCR: Quantitative real-time PCR
CMC: Sodium carboxymethylcellulose
