# Supplementary material for: Insights into the cellulose degradation mechanism of the thermophilic fungus Chaetomium thermophilum based on integrated functional omics
Source: Biotechnol Biofuels. 2020 Aug 12;13:143. doi: 10.1186/s13068-020-01783-z (PMC7425565; doi:10.1186/s13068-020-01783-z)
Supplement: Supplementary file 2 — Additional file 2: Supplementary Figures (S1–S6). [file 13068_2020_1783_MOESM2_ESM.docx]

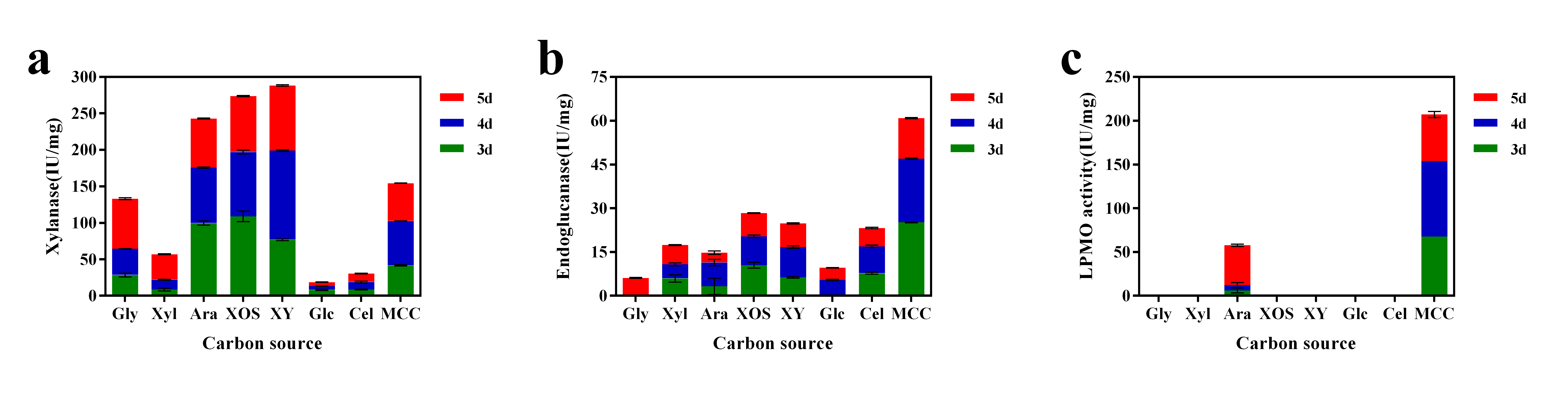


**Fig.** **S1** Specific activities (IU/mg) of *C. thermophilum* during growth on different carbon sources for 3-5 days. **a** Xylanase activity. **b** Endoglucanase activity. **c** LPMO activity.


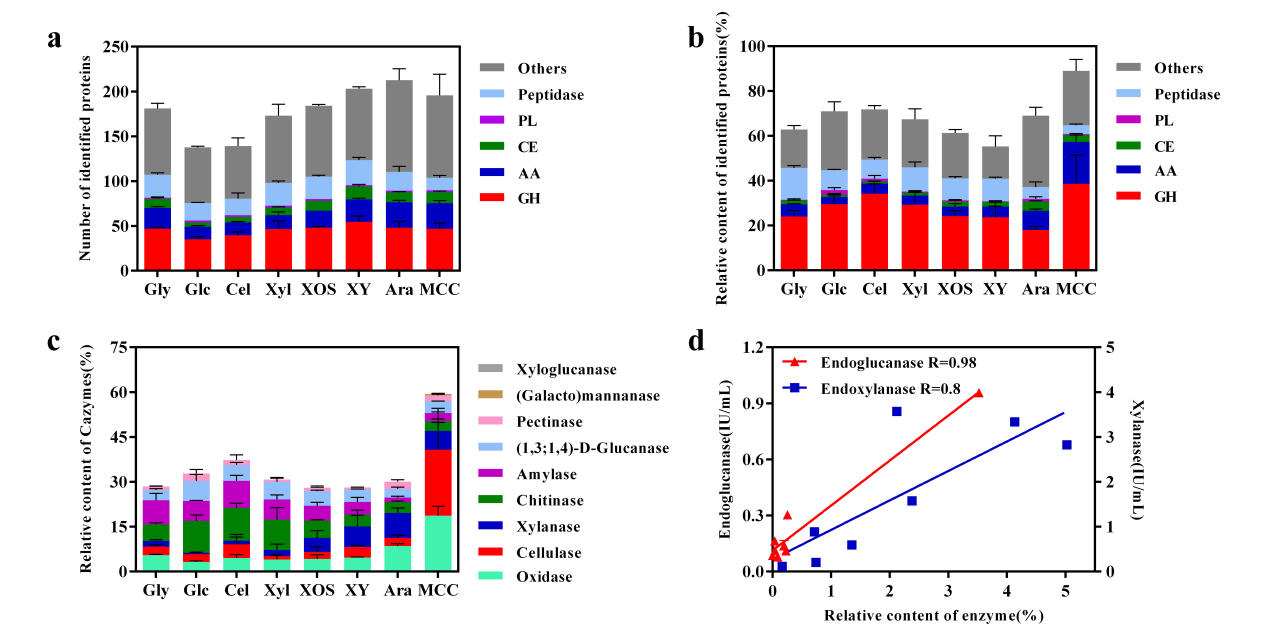


**Fig. S2** Detected CAZymes in secretomes of *C. thermophilum* grown on different carbon sources. **a** Protein species detected in the extracellular secretomes. **b** Protein content detected in the extracellular secretomes. **c** Selected enzymes related to polysaccharide degradation detected in the secretomes. **d** Correlation analysis between hydrolytic activity and relative content of corresponding enzymes.


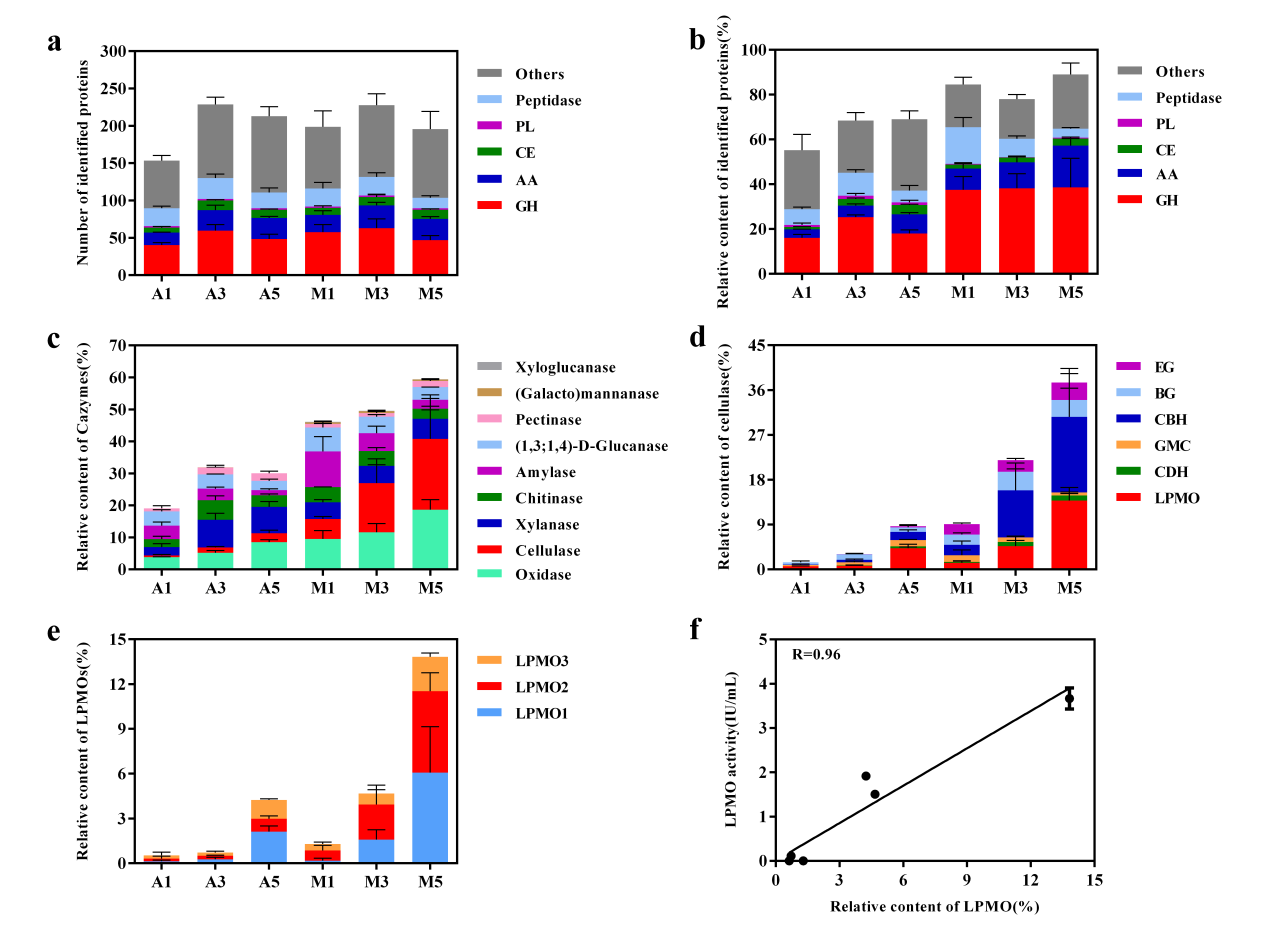


**Fig. S3** Detected CAZymes in secretomes of *C. thermophilum* during growth on arabinose and MCC at days 1, 3, and 5. **a** Protein species. **b** Relative content of proteins. **c** Relative content of selected enzymes related to polysaccharide degradation. **d** Relative content of enzymes related to cellulose degradation. **e** Relative content of the LPMO subfamily. **f** Correlation analysis of 2,6-DMP activity and relative content of corresponding enzymes.


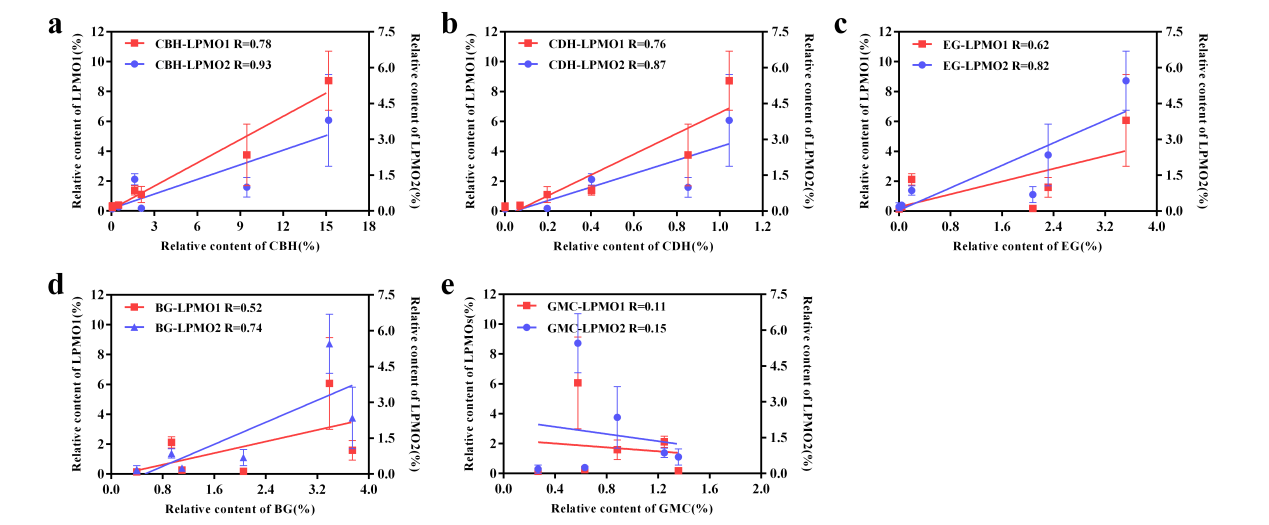


**Fig. S4** Correlation analysis between the relative content of the LPMO subfamily and corresponding enzymes. **a** LPMO and CBH. **b** LPMO and BG. **c** LPMO and EG. **d** LPMO and CDH. **e** LPMO and GMC.


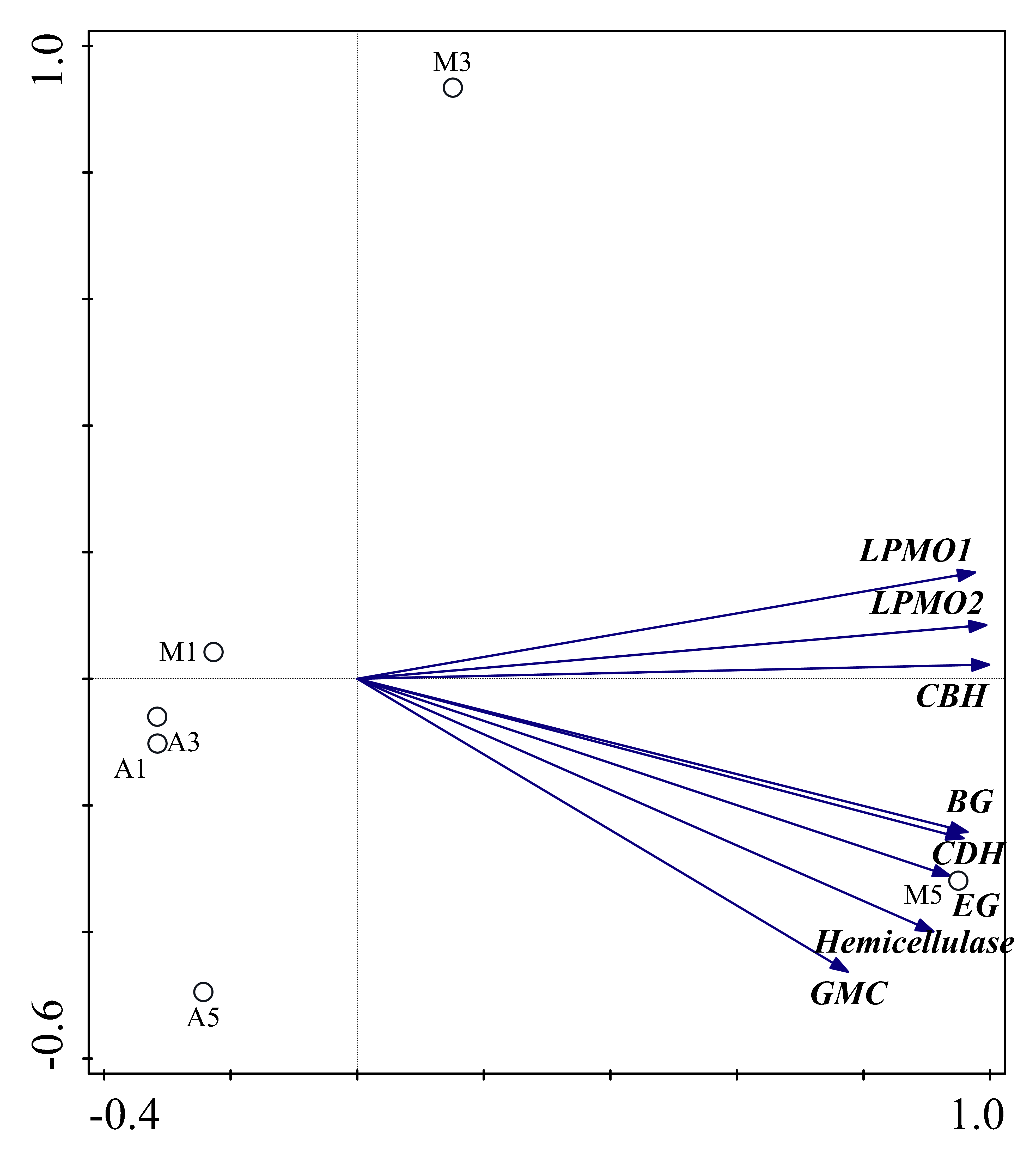


**Fig. S5** The principal component analysis of the transcript level of genes that related to the degradation of cellulose and hemicellulose. Positive associations between variables are identified when variables are clustered together (<90° angle), with smaller angles representing stronger associations.


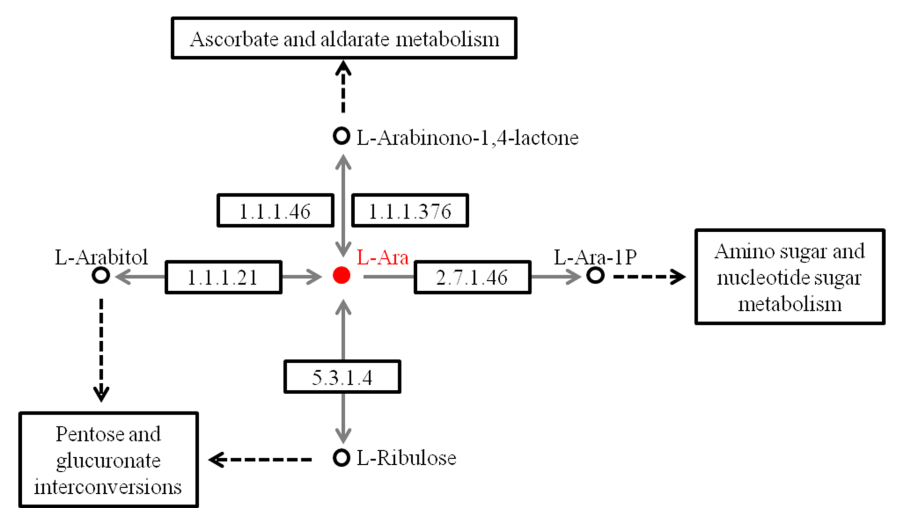


**Fig. S6** KEGG pathway of arabinose metabolism in *C. thermophilum.* The red circle indicates arabinose. The grey arrows indicate that enzymes needed in the pathway of arabinose metabolism are lacked.
